# The Traumatic Event in the Age of PTSD: Psychoanalysis, Neurology, and Literature

**DOI:** 10.1002/jhbs.70073

**Published:** 2026-11-03

**Authors:** Shaul Bar‐Haim

**Affiliations:** ^1^ Department of Sociology and Criminology University of Essex, Wivenhoe Park Colchester UK

## Abstract

The DSM‐III (1980) defined a traumatic event as a horrific event that is “outside the range of usual human experience,” such as genocide, war, a terror attack, or rape. However, by the publication of the DSM‐IV in 1994, there was a substantial expansion of the criteria for what a traumatic event could be, and who could be considered a victim of such an event. In the first decades of the 21st century, the discourse of trauma is ubiquitous in medical and cultural domains alike. Still, the traumatic event itself lost much of its currency, and trauma as such changed much of its meaning. This article documents the process by which this epistemological shift unfolded. The first part of the article presents the pedagogical intervention of literary scholar Shoshana Felman in 1984, in which she deliberately aimed to trigger highly emotional reactions in students by exposing them to recorded testimonies of Holocaust survivors. Emotional triggering stands, she believed, at the core of meaningful teaching of trauma in history. Felman was also a major figure in the second phase presented here, which took place in the mid‐1990s, with the fruitful and somewhat surprising collaboration between neurologically oriented clinicians such as the psychiatrists Bessel van der Kolk and Onno van der Hart the literary scholar Cathy Caruth and Felman. This collaboration contributed to the promotion of a new understanding of trauma in which triggers and flashbacks turned into the two main focal concepts in trauma discourse—and for PTSD to be popularized far beyond medical circles and academia, entering wider public domains and popular culture. This was the moment—as will be discussed in the last part of this piece—that a new “trigger culture” emerged (Ziv and Bar‐Haim, 2024). The notion that traumatic events from the past have long‐lasting and harmful effects on one's life has turned into an ongoing effort by individuals and the public to mitigate potential traumatic triggers in an unknown future.

## 1 Introduction

A main historical narrative in the history of mental health in the US is that since the 1970s, after three decades of dominating the American psychiatric establishment, psychoanalysis was gradually taken over by the new brain sciences and the pharma industry (Scull 2022). Different forces joined in the backlash against psychoanalysis in America in the late 1960s, as the demand for a radical shift in policy and medical approach to mental health arrived from different directions. The expansion of the psychiatric domain to cover more moderate mental conditions and family crises led insurance companies and the government to refuse reimbursement of methods which were not proven as “effective” enough, targeting specifically forms of dynamic psychotherapy or similar methods. Insurers, and later dissident psychiatrists alike, demanded clearer definitions of sickness and a shift away from a mode of thinking in which mental health problems were conceived as a spectrum to which everyone belonged—as is the perception in psychoanalytically oriented psychiatry. These forces were joined by the growing influence of anti‐psychiatry activists, influenced by the libertarian Thomas Szasz on the one hand, and New Left figures such as the Scottish psychiatrist R.D. Laing or activists in the Black Panther movement, on the other hand. They all argued that institutionalization is a form of incarceration and demanded to “liberate” or “emancipate” patients from their institutions to their own communities, never mind the fact that many of them were elderly or disabled, and that they had no one to care for them outside the hospital.

The growing costs of institutionalization and the increasing use of psychiatric drugs by psychiatrists provided the right conditions for the flourishing of the new “neo‐Kraepelinian” movement, characterized by what one historian called “descriptive psychiatry,” that is, “a psychiatry that limited itself to the description of the mentally ill and avoided speculation about etiology because it was unknown for almost all psychiatric diseases” (Decker 2007, p. 345). What started as an opposition to the principles of mainstream psychiatry in the 1960s by a few psychiatrists at Washington University in St Louis, such as Eli Robins, Samuel Guze, and George Winokur, turned in the 1970s into this movement, which dramatically changed the American mental health system. The “rebels” saw their full victory materialize with the publication of the DSM‐III of 1980, which indicated the abandonment of so‐called “speculative” theories such as psychoanalysis in favor of a symptom‐based diagnostic system (Mayes and Horwitz 2005). Mental illnesses and disorders were now defined through observable and measurable symptoms, rather than through the psychoanalytic emphasis on childhood experiences and their aetiological significance. The new manual was also an opportunity for a medical shift on several fronts with some major political implications. One of them was trauma, or more specifically, the introduction of a new medical category: Post‐Traumatic Stress Disorder (PTSD).

However, PTSD was not in itself anti‐psychoanalytic as other categories of the DSM‐III. In fact, some of its elements, like the acknowledgment of an “incubation period” between a traumatic event and the delayed onset of some post‐traumatic symptoms, are not alien to psychoanalytic thinking, and arguably originated in it. What some historians (e.g., Scull 2022; Horwitz 2018) described as a shift away from postwar Freudianism in 1970s American psychiatry was not immediately translated into a dramatic change. Moreover, trauma studies flourished since the 1980s in the social sciences and the humanities, where both PTSD and psychoanalysis only gained more cultural currency at the same time. The popularization of trauma as a cultural and historical category rather than only a medical one was gradual and included a major change in the definition of the “traumatic event” and the parameters for recognizing one. The DSM‐III defined a traumatic event as what “outside the range of usual human experience.” However, the popularization of trauma culture as we know it today arrived a decade later, with the DSM‐IV of 1994, which substantially expanded the criteria for what a traumatic event could be. The DSM‐IV taskforce dropped the requirement that the event would be “outside the range of usual human experience,” as it was no longer clear how to define a “usual” one. Moreover, with the new guide, doctors lost their sole authority to define what a traumatic stressor is. As Richard J. McNally, who was a member in the DSM‐IV taskforce pointed out, under the new criteria, “the [traumatic] event now was defined partly by the emotional response of the person,” that is, by the victim herself (McNally 2004, p. 3). In other words, the *democratization* of trauma started in the DSM‐IV because it allowed, for the first time, everyone to define any experience that they had as traumatic, and to attribute a wide range of psychosomatic symptoms as triggered by that specific event.

The early 1990s were also the historical moment when the discourse around PTSD shifted to some specific symptoms, and especially to victims' *flashbacks* (Jones et al. 2003). It was mainly the idea that flashbacks—always involuntary by definition—can be “triggered” by sensory reminiscences of the traumatic event which gained popularity in public domains and popular culture.^1^ PTSD was not only perceived as a threat for everyone—every difficult event for any individual could now be considered potentially traumatic—but the idea that some public spaces, and since the 2000s digital spaces as well, should be safer from trauma triggers gained ground. This new discourse that emphasized “safety” was, I will argue, the moment when the trauma discourse finally departed from psychoanalysis.

Alongside the evolving debates among psychiatrists about how to define PTSD, English departments turned in the 1980s into a hub for psychoanalytic thinking, especially about subjects such as collective trauma, memory, and witnessing. Moreover, disciplines such as English, History, and Film Studies closely engaged clinicians, neurologists, social scientists and artists in their research. This article aims to demonstrate the changing affinities between psychoanalysis and the new discourse of PTSD from the 1980s to our times by looking at the work of three influential literary critics in three different moments in time over the last four decades. First, I'll present the pedagogical intervention made by Shoshana Felman in 1984, in which she demonstrated how Holocaust testimonies can indeed *trigger* students to better understand and experience the epistemological position of the witness in a traumatic event. Triggering highly emotional reactions of students by exposing them to traumatic witnessing should be the core of meaningful teaching of the history of trauma, she would argue, in clear contrast to some trends in academia a few decades later.

The second moment of this article is the mid‐1990s, with the fruitful and somehow surprising collaboration between neurological‐oriented clinicians such as psychiatrist Bessel van der Kolk and psychologist Onno van der Hart and the psychoanalytically‐oriented literary scholars Felman and Cathy Caruth. The latter adopted some of van der Kolk's assumptions, by which the traumatic event is capable of destroying the survivor's linguistic capacity to narrate the event that created the post‐trauma at first place. The event, however, is “scored” by the body (as the title of van der Kolk's article and later best‐selling book *The Body Keeps the Score* indicates), and the only access that the traumatized subject may have to the traumatic experience is through her “flashbacks.” Flashbacks were perceived as a bodily re‐enactment of the traumatic event itself as if it is happening again there and then. The new focus on “flashback” indicates a shift in trauma discourse from a preoccupation around “what happened” to the victim (i.e., an attempt to make a coherent narrative out of the facts, as much as they are available) to what is it that being reenacted by the flashback (i.e., the facts as being told by the victim's body).

This was a shift that led to the popularization of PTSD far beyond medical circles and academia into other public domains and popular culture, and to the emergence of a new “trigger culture” (Ziv and Bar‐Haim 2024) that started to take place—one in which young people were asking to create “safe” spaces where they can be able to recognize potential trauma triggers and avoid them. I conclude the article with the criticism of Jack Halberstam, who called, in a couple of publications in the 2010s, for abandoning the current understanding of ordinary life events, or of representations of catastrophes, as potentially traumatizing, and to go back to some old Freudian perceptions of the *psyche* in which subjects (in his case, the students in his classroom) are far more resilient than they themselves can believe.

## 2 The Road to PTSD

As I discussed elsewhere (Ziv and Bar‐Haim 2024), PTSD was first included in the DSM‐III in 1980, following a long campaign by two main survivor groups: Vietnam veterans and Holocaust survivors. Although the historical contexts of these survivor groups differed, both the American and West German psychiatric establishments were reluctant to acknowledge the Holocaust and the Vietnam War as direct causes of the poor mental health experienced by the survivors. In the German case, psychiatrists relied on Freudian theories to argue that the horror of the Holocaust alone could not sufficiently traumatize victims (Herzog 2016). Some psychiatrists even went so far as to suggest that childhood experiences and family dynamics, rather than the horrors of the Holocaust itself, might have predisposed certain individuals to psychological suffering. As the psychoanalyst Kurt Eissler scathingly described it in 1963: “The murder of how many of one's children must one be able to survive asymptomatically in order to be deemed to have a normal constitution?” (cited in: ibid, 107). Some of those cases were fought for in German courts for decades, and the courage of a few psychiatrists on both sides of the Atlantic, such as Kurt Kolle and Eissler, who advocated for changes in psychiatric categories to better reflect historical realities, was crucial in postwar debates on compensation and in the eventual recognition of PTSD.

The case of Vietnam veterans was historically different, but they suffered from similar reluctance on the part of the establishment to recognize their suffering as post‐traumatic (Scott 1990; Jones and Wessely 2006). Many veterans did not initially show post‐traumatic symptoms, which often emerged only a few years after their return from Vietnam. Consequently, the American establishment was reluctant to acknowledge the connection between military service in Vietnam and subsequent psychological suffering. Moreover, many veterans suffered in the 1970s from a range of mental struggles that manifested as difficulties readjusting to civilian life, finding jobs back home, as well as addiction and anti‐social behaviour. Whether these problems were more prevalent among Vietnam veterans than among other groups or not is still under debate among historians. However, the image of the violent, anti‐social veterans remained fixed in American culture in the decades to come. Furthermore, the neglect of veterans was not unrelated to the public perception of the war as a colossal defeat. Americans wanted to put the war behind them.

Thus, the engagement of activist‐psychiatrists Chaim Shatan and Robert Jay Lifton in the Vietnam Veterans Against the War movement was critical in making the suffering of neglected veterans visible, advocating for them, and seeking to politicize the category of trauma in a way that emphasized the war as the cause for the veterans’ poor health. In fact, it was Shatan who coined the term “post‐Vietnam syndrome” in 1972—a term that was in many respects a precursor to PTSD, although, as Abu El‐Haj (2022) argues, PTSD was a depoliticised concept compared with “post‐Vietnam syndrome”, which explicitly linked veterans’ psychological suffering to the war. Nonetheless, we can see how the road to PTSD was no less political than medical: the aetiology of post‐traumatic symptoms had to be understood in relation to political, and even geopolitical, events, shifting the focus from individuals who allegedly suffered from poor psychological development or inborn vulnerabilities to past (and often long past) traumatic events.

The introduction of PTSD was an important victory for army veterans and Holocaust survivors, with huge further implications for many other forms of trauma—especially domestic and sexual abuse—but it also changed dramatically the way we understand the traumatic event itself. As Fassin and Rechtman (2009) have written, “[t]he clinical signs of PTSD remained those of classic traumatic neurosis, but the status of the traumatic event had fundamentally altered, becoming the necessary and sufficient etiological agent” (p. 86). If in the past, the assumption was that the group as a whole was “healthy” but some individuals in it were weaker and more vulnerable to trauma than others, now the assumption has become almost the opposite, namely that people are unlikely to survive horrific events such as genocide, war, terror attacks, domestic abuse or major individual adversities without developing any post‐traumatic symptoms. As Lifton (1993) puts it, “the posttraumatic stress disorder is a normal adaptive process of reaction to an abnormal situation” (p. 12). However, those traumatic events remained underdefined and very often unidentified: they could be understood as traumatic only retrospectively, after the emergence of post‐traumatic symptoms.

As will be discussed in the last part of the article, we now have some reasons to think that another shift in the discourse of trauma occurred in the last two decades, namely that currently the focus is not necessarily on the *event* itself but on “traumatic triggers” that may or may not ignite post‐traumatic reactions and specific symptoms. The effort now is to prevent those symptoms in the first place by creating environments in which reminiscences of the traumatic events are unlikely to appear by making public spaces as “safe” as possible, which means in many cases for role‐holders in public spaces to second‐guess what can be a trigger for unknown audiences. If in the past the discourse around trauma focused on horrific events that had already occurred, now the effort in some circles is to prevent unpredictable mental symptoms or worse from appearing in the future.

## 3 The Traumatic Event in the Classroom

The DSM‐III was designed throughout the 1970s as a new non‐Freudian symptom‐based diagnostic model. Indeed, one of the main goals in the 1970s of the special taskforce for the making of the DSM‐III, and its influential chair, Robert Spitzer, was to discredit psychoanalysis as a reliable diagnostic theory. By doing that, they paved the way for the introduction of a new diagnostic system for mental illnesses and disorders—one which could not be open to interpretation and could not be based on the patient's past or circumstances, but only on the patient's current behavior, and their bodily psychosomatic symptoms at the present. However, while the DSM‐III was supposed to simplify the diagnosis of mental health problems, in fact it opened the door for all sorts of new medical categories such as the five new types of eating disorders and eight new psychosexual disorders. Thus, for example, the hitherto one type of what was known as “phobia neurosis” was now replaced by agoraphobia, social phobia and single phobia, while none of them was associated with the N‐word, that is “neurosis.”

The story of how PTSD got included in the DSM‐III—probably more of a political compromise that was forced on Spitzer rather than his full endorsement—is well documented, and cannot be discussed here in detail (Scott 1990; Horwitz 2018; Abu El‐Haj 2022). However, for our purposes it is important to highlight that PTSD was an outlier disorder in the DSM‐III from the outset. On the one hand, it was a diagnosis that was heavily based on patients' symptoms, as the new medical framework of the DSM‐III demanded. On the other hand, however, it was also based on the idea that traumatic events may have an “incubation period” in one's mind before they can be fully converted into a psychosomatic disorder, and that in many cases PTSD symptoms have delayed onset. The reliance on one's past in defining one's trauma was heavily invested in psychoanalytic ideas even if the discourse was no longer fully Freudian.

PTSD is considered today—and for some real reasons—as a non‐psychoanalytic diagnosis, but it wasn't always the case. Indeed, some of the major clinical concepts that emerged from PTSD, such as “flashbacks” and “triggers,” are quite open to psychoanalytic interpretations. Psychoanalysis provided the archetype model for many of the 20th century theories of trauma, in which memories of a horrific event are seeded in one's mind, then repressed for a long period of incubation, before erupting as a crisis of post‐traumatic psychosomatic collapse. Indeed, Freud believed that traumatic events can be transmitted intergenerationally not only on the individual level, but also among collectives, as was suggested first in *Totem and Taboo* in 1913, and then again in his monumental last project, *Moses and Monotheism*, which was written throughout the 1930s, but was published only in 1939, just after Freud managed to escape with his family to London.

We should bear in mind that it is not by chance that PTSD started to take over as the organizing framework of trauma at the same time that academics, artists, and public intellectuals in Europe and the US got increasingly preoccupied with questions about collective memory, and the status of survivors' testimonies in shaping what some sociologists will describe later as the “cultural trauma” of European Fascism and its victims, mainly but not only the Jews (Alexander 2004).^2^ Most public attention was given in the 1980s and 1990s to Holocaust survivors, who started to be given different main platforms for telling their story of the genocide that they survived (Hirschberger 2018; Wieviorka 2006; Felman and Laub 1992). One reason for what scholars called the “memory boom” of the 1980s (Winter 2006) was the introduction of new, more available, and easier to operate audio‐visual technologies, which made interviews and testimonies with survivors possible on a much larger scale (Pinchevski 2012). One of the main platforms for collecting testimonies was the Fortunoff Archive at Yale University.

Initiated in 1979 by the producer Laurel Fox Vlock and the psychiatrist and Holocaust survivor Dori Laub (both lived then in New Haven, Connecticut), the Fortunoff Archive was a first of its kind audiovisual collection of Holocaust survivors' testimonies, using the then‐pioneering technology of video recording. By 1981, they managed to produce and record over 200 testimonies, and ever since then the number of recorded testimonies increased to thousands, which were widely researched by Holocaust scholars. One of the first to use them in her classroom was the literary and psychoanalytic scholar Shoshana Felman.

In her article “Education and Crisis, or the Vicissitudes of Teaching” published in the journal *American Imago* in 1991, she described the experimental graduate course that she had convened in 1984 in her home institution, Yale University, under the title “Literature, Psychoanalysis and History” (Felman 1991).^3^ The first half of the course may seem like a standard module in modernist culture, with an exploration of the concept of testimony by reading the works of Camus, Dostoevsky, Celan, and Freud. The second half of Felman's module, however, was dedicated to bridging the gap between the literary text and the documented witnesses—or what Felman called “life testimonies”—by watching together two video testimonies of Holocaust survivors, taken from the then new Yale archive.

Felman organized the screening of two survivors' testimonies: Helen K. and Menachem S.^4^ Both survivors escaped as children from the fate of their families—all their families got murdered by the Nazis—and both of them eventually managed to build new lives and, as they testified, to find new meanings to those lives, after losing in the Holocaust everything but themselves.

However, Felman's main interest in screening the testimonies was not historical as such. The recordings provided her the platform for demonstrating to her students the transformative power of the traumatic event once it turns into a testimony—transformative not only for the survivors but also for their audience. The screening of the testimonies created a crisis in class that Felman wholeheartedly embraced. The crisis emerged between the screening of the first and second video. The first video was of Helen K., who lost in the camps “her father, her mother, her youngest brother, her sister‐in‐law, and their baby (the last three [were] dying in her presence)”—but then found after the war her husband, who was “arranged” to her by her father, still in the Ghetto when she was 16.

Helen K.'s testimony shocked the students so profoundly that it created a form of collective mental breakdown, or arguably a “mass hysteria” that wasn't anticipated by Felman but was retrospectively welcomed by her:

The tapes were screened in the informal privacy of an apartment, with the students sitting on the carpet, all over the floor. During the screening some were crying, but that in itself is not an unusual phenomenon…. I realized that something strange was going on when I started getting phone calls from the students at my home at all odd hours of the night or of the day, to talk with me about the session, although they did not quite know what to say: since I felt that what they needed was the contact, I simply was receptive to their calls, and listened. As I later learned from my colleagues, the students of my class who met in other classes would only talk about the session, and not about the topic of the other class. Friends and roommates of my students later wrote me letters to tell me of the interest they had developed in my class, by virtue of their having had to listen to my students, by virtue of their having become, as one letter puts it, the “coerced listeners” to these outside proceedings of the class and to my students' frantic talk… They were obsessed. They sought out each other and yet felt they could not reach each other…. They felt alone, suddenly deprived of their bonding to the world, and to one another.

(Felman 1991, p. 59)

Felman's account included a statement that she gave to the class after the first screening and after realizing that the well‐being of her students is at stake. She realized that she needed to intervene, but she also wanted to make sure that the second screening—the testimony of Menachem S.—would be possible at all. It seems like she did not even contemplate the idea of not going ahead with the second testimony given the way it affected her students' well‐being. For Felman, the show had to go on, come what may. Felman recognized that some students felt that they were having a breakdown, but even among them it wasn't always felt as only a destructive experience but also a formative one, as if they understood that an educational goal can be registered meaningfully in their mind only by allowing themselves to be *triggered*.

“Triggering” was not yet part of the discourse in 1984, but interestingly Felman told her class that “[w]hat your responses most of all conveyed to me was something like an anxiety of *fragmentation*. People talked of having the feeling of being ‘cut off’ at the end of the session” (p. 61). Fragmentation will become a few years later a central concept to the conceptualization of the traumatic event as a dissociative mechanism of the victim (van der Kolk and Fisler 1995). Felman, however, felt like it is the *anxiety* of “fragmentation” that takes place, as if students were shocked that they had to go through such a potentially “fragmenting” experience without any “trigger warning” many years before those warnings were even an option. She quoted one student who told her that “until now and throughout the texts we have been… talking about ‘the testimony of an accident’. We have been talking about the accident—and here all of a sudden the accident happened in the class, happened to the class. The accident passed through the class” (Felman 1991, p. 62).

Felman herself concluded that “as the accidental and yet generally valid lesson I have learned from that class, that teaching in itself, teaching as such, takes place precisely only through a crisis” (Felman 1991, p. 67). The crisis was described as an accident, which resonates with a then new PTSD‐led trauma discourse in which the single traumatic event (for example, an “accident”) is a stressor in the formation of a post‐trauma condition (rather than a weak, vulnerable or troubled psyche, as it was hitherto described with those terms under the Freudian paradigm). However, one may feel a strong unease in the a‐historical description of the trauma caused by the Holocaust as an “accident.” Moreover, some of the main criticisms against PTSD in the decades to come were related to this focus on single traumatic events rather than on enduring ongoing traumatic realities, which led researchers to come up with new clinical definitions or descriptions such as Complex‐PTSD (Herman 1992), or “insidious trauma” (Brown 1991).

Felman made a direct analogy between an analyst and a teacher. She compared herself to a psychoanalyst who “serves as witness to the story of the patient, which he then interprets and puts together, so did I return to the students—in their own words—the narrative” (Felman 1991, p. 69). She was suggesting that the educational vocation is not totally dissimilar to the psychoanalytic one. They are both about witnessing, and in this case, Felman was bearing witness not only to the history of Holocaust' survivors but also to her students' mental breakdown. But even if therapy and education are both occupations of and about *care for the suffering other*, Felman never crossed the line of becoming her students' therapist rather than their teacher. Presenting the testimonies was more important to her than her students' well‐being. Helen K. and Menachem S. told their story, and the students had to listen. The class itself turned into what one may define as a *non‐safe* pedagogical space, which Felman may claim to be, in this case, a necessary condition of the pedagogical process.

The seminars took place in 1984, between the publication of DSM‐III and DSM‐III‐R. The DSM‐III defines a traumatic “stressor” as an event “that would evoke significant symptoms of distress in almost everyone.” The DSM‐III‐R, published in 1987 expended the definition of traumatic stressors by including the witnessing or hearing about a traumatic event involving one's close relatives and friends. This was a major change in the sense that it could define people as suffering from PTSD even if they didn't experience a traumatic event firsthand. However, the event itself was still defined as the exceptionally horrific one, and it was not for everyone to define traumatic events subjectively, as would be allowed a few years later by the DSM‐IV of 1994. In that sense, the pedagogical experiment of Felman was in line with the PTSD‐III‐R of 1987 in which the event itself—the Holocaust—was undoubtedly traumatic, but this event could be traumatizing not only for the direct survivors, but also for “witnesses” and other people who didn't experience the event firsthand. For example, the students in Felman's class.

## 4 Flashbacking the Traumatic Event

The 1990s were a decade of surprising encounters and collaborations between psychoanalytically oriented literary scholars like Felman and her close colleague Cathy Caruth, and trauma researchers and psychiatrists with neurological and biological orientation, such as Bessel van der Kolk and Onno van der Hart. Since the late 1980s, they revived a hitherto overlooked tradition of trauma, namely that of the French psychologist Pierre Janet (1859–1947) (van der Kolk and van der Hart 1989).^5^ For Janet, as for many of his contemporaries in nineteenth‐century French psychiatry, trauma was a memory disorder. His fundamental distinction, which was later adopted by late twentieth‐century psychiatrists, was that between “narrative memory” and “traumatic memory”. The former consists of recollections integrated into a coherent account of the past, which the subject can narrate to herself and to others. The traumatic memory, however, “occurs automatically in situations which are reminiscent of the original traumatic situation. These circumstances *trigger the traumatic memory*” (Janet 1919, cited in: van Der Kolk and van der Hart 1991, p. 431 emphasis added).

By bringing Janet's theories back to the forefront of trauma research, van der Kolk and his colleagues advanced the idea that “it is in the very nature of traumatic memory to be dissociated” (van der Kolk and Fisler 1995, p. 518) – that is, dissociation is the failure to integrate traumatic memories into one's narrative memory.^6^ According to van der Kolk et al., dissociative states in trauma victims involve “alterations in the experience of time, place, and person, which conferred a sense of unreality on the event as it was occurring” (1996, p. 313). As traumatic memory, according to this model, cannot be verbally recounted by the victim, it may instead be expressed through bodily re‐enactments of the trauma, or what we now call “flashbacks.”^7^ Sensory signals associated with the traumatic event have the power to activate – or to “trigger” – a flashback. However, because this activation is unpredictable and beyond the victim's control, the triggers themselves become sources of anxiety. As Janet observed, the traumatized “suffer…from a phobia for the traumatic memory” (van der Kolk & van der Hart 1991, p. 447).

By the mid‐1990s, van der Kolk was endorsed and supported by the literary and psychoanalytic scholar Cathy Caruth, who presented at the time her own theory of trauma. For Caruth (1996), the traumatic event is by definition dissociated in the memory of the survivor, as the catastrophe was so damaging and horrifying that it destroyed all forms of language by which one can narrate their own story. What remains are “flashbacks” which are non‐lingual or “nonrepresentational,” in Caruth's terms (p. 115)—bodily sensory images of the event itself. The “literal” reminiscences intrude on the survivor's mind and reenact the traumatic events in ways which are not simply representational. Indeed, the reenactment, Caruth argued, is being bodily experienced by the victim as the event *itself*. The association of Caruth with psychoanalysis did not stop the leading psychiatrists—somehow identified later by some as anti‐psychoanalytic—from collaborating with her. Van der Kolk and Onno van der Hart published their chapter in Caruth's influential edited volume, *Trauma: Explorations in Memory*, alongside psychoanalytic literary scholars such as Harold Bloom and Shoshana Felman, and indeed psychoanalysts such as Dori Laub (Caruth 1995).

In her seminal book *Trauma: A Genealogy*, historian Ruth Leys delivered a devastating critique of both van der Kold and Caruth, beginning by asking “what are we to make of the fact that modern neurobiology and certain versions of poststructuralism share virtually the same account of psychic trauma?” (Leys 2000, p. 229). Part of her criticism is valid and even important, but much of it is about proving that Caruth is not Freudian enough or not Freudian at all, even if she pretends to be so. Leys also accused Caruth of misreading Freud and of adopting what she thought was van der Kolk's anti‐psychoanalytic approach, although, as we have seen, it is unclear if van der Kolk was (or still is) anti‐psychoanalytic at all. Leys devoted parts of her chapter to showing the influence of Felman, and Felman's mentor at Yale, the philosopher Paul De Man, on her positions on trauma and her reading of Freud. Felman (2002) replied to Leys in her book the *Juridical Unconscious* in a footnote of not less than nine pages in which she defended Caruth, as well as her reading of what she called the “mature” Freud—perhaps referring here mainly to his *Beyond the Pleasure Principle*—in which the idea of the literality of the flashback is more plausible (Felman 2002, pp. 173–182).

Leys, however, is right to identify the emergence, in the 1990s, of a new network of literary scholars psychiatrists, psychologists and feminist therapists such as some of the figures mentioned above, who aimed to integrate psychoanalysis into the increasingly influential field of neuroscience, in which flashbacks were perceived as the defining characteristic of the post‐traumatized survivor (see for example, Lanzmann et al. 1991). Flashbacks were now acknowledged as the core of suffering and the main symptom for the traumatized patient under the framework of PTSD, but it was also being used by theorists as perhaps the only epistemological access for the clinician to know “what really happened” in the traumatic event—the factual core of the event that cannot be gained otherwise, as the traumatic memory was perceived as incapable of any narration. Therefore, educationalists such as Felman and Caruth kept thinking of the “flashback” as a model for reenacting the catastrophic event which in itself was crucial for maintaining the injunction of remembering. Indeed, it was a historical moment where psychoanalysis and neuroscience provided each other languages that each discipline was missing.

## 5 The Age of “Trigger” and the Decline of the “Traumatic Event”

In 1992, Judith Herman published her now canonical article “Complex PTSD: A syndrome in survivors of prolonged and repeated trauma,” in which she suggested a new syndrome—the Complex PTSD—characterized by “pleomorphic symptom picture, enduring personality changes, and high risk for repeated harm, either self‐inflicted or at the hands of others” (Herman 1992, p. 387). It is mainly a situation of “captivity, which brings the victim into prolonged contact with the perpetrator, creates a special type of relationship, one of coercive control” (p. 378). The difference between PTSD and the then‐newly proposed diagnostic criteria is that C‐PTSD does not refer to a specific traumatic event but to life experiences of an enduring “prolonged victimization.” The idea that trauma cannot be limited to looking backward into a specific event in one's life but into a whole traumatic ecosystem led to new ways of theorizing trauma as a living experience which is based on duration and not on a singular event or some specific events. A decade later, van der Kolk, coined a different term, “developmental trauma” (partly based on Bowlbyan ideas), for describing the developmental damage occurred in children with “insecure attachment” caused by their dysfunctional parents. For van der Kolk, this new category of developmental trauma was necessary because “the diagnosis PTSD is not developmentally sensitive and does not adequately describe the impact of exposure to childhood trauma on the developing child” (van der Kolk 2005). These two definitions, together with other emerging concepts of trauma such as Maria Root's “insidious trauma” (see below), rejected a focus on the traumatic event as a singular, non‐linguistic, and inaccessible occurrence, and instead promoted a new framework for understanding trauma as a duration shaped by a whole life experience of hardship and suffering.

But the critique was not only about defining what can be considered a traumatic living experience according to its duration, but also about the very core definition of the DSM‐III of a traumatic event as an event that takes place “outside the range of usual human experience,” citing combat, rape, bombing, torture, and death camps. In the DSM‐III‐R of 1987, the definition was expanded to “serious threats to one's life or physical integrity,” as well as to one's close relatives and friends. But the bar was still so high as to exclude all sorts of ongoing forms of violence and adversities that cannot be as explicit as the mentioned examples. The challenge to this narrow definition of trauma came from feminist psychotherapists and psychologists who argued in the late 1980s that the high threshold embedded in such definitions excluded large numbers of women, girls, and other marginalized groups, whose experiences of trauma were therefore not acknowledged. Psychologist Maria Root, coined the term “insidious trauma,” which was further popularized by her colleague Laura S. Brown, who defined it as “the traumatogenic effects of oppression that are not necessarily overtly violent or threatening to bodily well‐being at the given moment, but which do violence to the soul and spirit.” Thus, they both argued that “for all women, living in a culture where there is a high base rate of sexual assault, and where such behavior is considered normal and erotic by men as it is in North American culture, is an exposure to insidious trauma” (Brown 1991, p. 128). She concludes by criticizing the already dominating paradigm of PTSD, which overlooks the question of “how many layers of trauma are being peeled off by what appears initially to be only one traumatic event or process?” (p. 131). The idea that trauma is a physical reality that is based on psychical “layers” complemented other approaches that now focused on the intergenerational dimension of trauma, passing patterns of harm and suffering from one generation to another. The intergenerational trauma perspective developed initially from the study of second and third generations of Holocaust survivors (Bar‐On 1995; Yehuda and Lehrner 2018), before being extended to other collectives who were victims of genocide and mass violence, as well as adopted by feminist clinicians as a main framework for understanding intergenerational patterns in victims of sexual and domestic abuse and violence.

However, the evolving definition of PTSD not only changed the way we understand trauma, but also helped trauma to become a meta‐concept to be used in larger domains of mental health more generally. Rather than medicalization of everyday life, we can now see the *traumatization* of everyday life, where people are increasingly encouraged to diagnose themselves as suffering from post‐trauma. Some scholars described our current willingness to accept living with post‐trauma symptoms as the “new normal” (Elsaesser 2021; Koch 2021). We can particularly see how concepts about collective trauma have been dramatically expanded, allowing people to claim PTSD mainly by being part of a group and not necessarily through a personal traumatic experience. This expansion of the claim for the post‐traumatic experience accelerated under a psycho‐medical model in which “flashbacks,” or other unpredictable psychosomatic symptoms, are perceived as the main terror of victims—and the notion that they can be “triggered” by some sensory contents that may immediately bring to life the traumatic past in a form of reenactment. As one cannot necessarily predict what event may or may not register itself as traumatic in one's mind, the outcome is an ongoing cautious from post‐traumatic symptoms being triggered by identified or unidentified—well‐remembered or not remembered at all—event or events that happened in the past. But the event itself became almost less important than its registration in one's traumatic memory. The lack of any sense of objective criteria for what could be counted as a traumatic event made everyday life a minefield full of potential trauma and suffering for the future.

The emergence of the *trigger‐flashback* logic was also the historical moment when “trigger warnings” started to take a much more central place in public life and culture. The earliest use of trigger warnings appeared in online forums and social media, where they served to alert readers about content that might provoke a harmful psychological reaction. Their adoption, both online and offline, initially featured within feminist, queer, anti‐racist, and other progressive subcultures, before turning into a widespread practice, mainly in North American academia. Within the academic context, Bryce et al. (2023) define trigger warnings as “explicit statements that alert a group of learners that certain content explored or discussed in a learning environment may contain potentially distressing material” (p. 2883; see also Gerdes 2019, P. 5).^8^

As Ziv (2018) argues, the growing demand for trigger warnings reflects a wider transformation in academic culture, in which students are increasingly treated as psychologically vulnerable and come to perceive themselves as such. In an article from 2017, queer theorist and a main progressive voice *against* trigger warnings, Jack Halberstam, told an anecdote from their own teaching in a course on the Holocaust: “students were appalled and shocked on the first day of class when I showed clips from Alain Resnais's classic 1955 film Night and Fog. The now all‐too‐familiar imagery of bodies being shoveled into shallow graves, of mounds of rotting and emaciated flesh, was more than some students in the class wanted to see, and they left the room.” But Halberstam's position was that “the clips from Night and Fog need to be seen without warnings ahead of time in order to allow the students to grapple with their own shocked reactions before delving into the history of the representation of genocide” (Halberstam 2017, p. 540). They refused to accept the “projection of the student as a fragile organism with no intellectual immune system and a minefield of a psyche that may explode into pieces at any moment” (p. 539).

Searching back for historical models where the mind was perceived as more agile and resilient, Halberstam revives the Freudian “mystic writing pad.” In a well‐known paper, Freud (1925) compared the conscious and the unconscious to a writing device that was popular at the time (and still exists today), known as the “mystic writing‐pad.” The classic version of the writing pad had two layers: the lower one, made of soft wax, and the upper one, a thin sheet of celluloid on which writing takes place. What is written with a stylus on the celluloid can be instantly erased by lifting the top layer, yet the impression remains on the almost invisible wax—just as memories are stored in the unconscious.^9^ For Halberstam's students, however, the internalization of troubling or even traumatizing images no longer operates according to the logic of the mystic writing pad, as memories are no longer stored and repressed, but remain on the surface, always ready to be triggered, like “a live wire sitting in the psyche waiting for a spark” (Halberstam 2014). Rather than trauma in its modern sense, that is, a bodily and mental reaction of the traumatized subject to a catastrophic event *after*—and often indeed long after—the event ended, we are now facing a new trigger‐based model of trauma which often looks much more like an “anticipatory anxiety” of an unknown future.^10^

Is Halberstam's “back to Freud” only a nostalgic, arguably reactionary, view of the 20th century psyche as resilient and stronger than ours—almost a desire of an English professor to make America Freudian Again; or perhaps a genuine concern about the capacity of young people, and indeed all people, to endure hard realities and their representations as they are? Or perhaps it is a nostalgic call for the past, but not a call to Freud of 1925 and his “mystic writing‐pad” but to Shoshana Felman of 1984, when triggering graduate students in U.S. elite institutions was considered as the right pedagogical thing to do.

## Conflicts of Interest

The author declares no conflicts of interest.

## Data Availability

Data sharing is not applicable to this article, as no new data were created or analyzed in this study.

## Endnotes

1 By “trigger” I mean “a sensory reminiscence of the traumatic event that has the potential to revive the event in the survivor's memory and thus activate a set of symptoms.” (Ziv and Bar‐Haim 2024, p. 437)

^2^
In this context, it is also important to highlight Claude Lanzmann's monumental documentary Shoah, widely regarded as one of the most influential documentaries of the twentieth century, which premiered in 1985.

3 The article was later included as a chapter in the well‐known volume edited by Dori Laub and Felman, *Testimony: Crises of Witnessing in Literature, Psychoanalysis, and History* (1992).

4 The testimonies are available at: https: https://web.library.yale.edu/news/2009/09/helen‐k‐edited‐testimony‐hvt‐8035, https://web.library.yale.edu/news/2009/09/menachem‐s‐edited‐testimony‐hvt‐8063.

5 The following discussion draws on Ziv and Bar‐Haim (2024).

6 Janet was known in the history of psychiatry as one of Freud's many rivals, but the extent to which their theories of memory are fundamentally different is still contested. See, for example, Sanfelippo and Dagfal (2020). Compare also van der Kolk (1989) with *Beyond the Pleasure Principle* (Freud 1920), where repetition‐compulsion lies at the core of the traumatic experience. It is hard to overestimate the influence of van der Kolk on perceptions of trauma among professionals and the public alike (his 2014 book *The Body Keeps the Score* is one of the most prominent non‐fiction bestsellers of the twenty‐first century so far). However, some leading cognitive psychologists have repeatedly criticised van der Kolk's views on dissociated or repressed memory. See, for example, McNally (2024).

7 According to the OED, “flash‐back” (with the hyphen) first appeared in 1916 in a cinematographic context to describe a return to a previous action or event in a film. In the 1960s and 1970s, some researchers started to use the concept to describe experiences of people who used psychedelic drugs (also known as “hallucinogenic drugs”), especially LSD (Frankel 1994). But in difference from later understanding of flashbacks as an accurate and literal representation of real events, psychedelic drug researchers believed that flashbacks are partly imaginative and cannot be taken at face value.

^8^
The debate over the effectiveness of trigger warnings in reducing traumatic reactions, as well as their social and cultural implications, has been ongoing for many years, not only in academic circles but also in the general press and among the wider public. For a selection of the extensive literature for and against trigger warnings see, for example, Lukianoff and Haidt 2015; Manne 2015; George and Hovey 2020; Bower 2025; Pratt et al. 2025. 

9 For the surprising history of the “mystic writing‐pad” and its role as a leading tool in early 20th century industries and the army, see Williams (2024).

10 Davies (2025) recently framed our current times as the “age of anxiety” shaped by “a narrative surrounding the state of overwhelming futurity that is a consequence of economic conditions as much as psychic ones” (p.15). The outcome of this new futurity—not dissimilar in its temporality to the one I described here—is a new “anticipatory selfhood.” By that, Davies means that people of all backgrounds struggle with ongoing deteriorating mental health and material resources while keeping themselves prepared for the worst yet to come in the form of environmental collapse, global wars, and digital revolutions.
